# Reduced cerebrospinal fluid ethanolamine concentration in major depressive disorder

**DOI:** 10.1038/srep07796

**Published:** 2015-01-15

**Authors:** Shintaro Ogawa, Kotaro Hattori, Daimei Sasayama, Yuki Yokota, Ryo Matsumura, Junko Matsuo, Miho Ota, Hiroaki Hori, Toshiya Teraishi, Sumiko Yoshida, Takamasa Noda, Yoshiaki Ohashi, Hajime Sato, Teruhiko Higuchi, Nobutaka Motohashi, Hiroshi Kunugi

**Affiliations:** 1 Department of Mental Disorder Research, National Institute of Neuroscience, National Center of Neurology and Psychiatry, Kodaira, Tokyo, 187-8502, Japan; 2 Translational Medical Center, National Center of Neurology and Psychiatry, Kodaira, Tokyo, 187-8551, Japan; 3 Department of Psychiatry, Shinshu University School of Medicine, Matsumoto, Nagano, 390-8621, Japan; 4 Department of Psychiatry, National Center Hospital, National Center of Neurology and Psychiatry, Kodaira, Tokyo, 187-8551, Japan; 5 Human Metabolome Technologies, Inc., Tsuruoka, Yamagata, 997-0052, Japan; 6 National Center of Neurology and Psychiatry, Kodaira, Tokyo, 187-8551, Japan; 7 Department of Neuropsychiatry, Interdisciplinary Graduate School of Medicine and Engineering, University of Yamanashi, Chuo, Yamanashi, 409-3898, Japan

## Abstract

Amino acids play key roles in the function of the central nervous system, and their alterations are implicated in psychiatric disorders. In the search for a biomarker for major depressive disorder (MDD), we used high-performance liquid chromatography to measure amino acids and related molecules in the cerebrospinal fluid (CSF) of 52 patients with MDD (42 depressed and 10 remitted; DSM-IV) and 54 matched controls. Significant differences were found in four amino acid concentrations between the depressed patients and controls. After Bonferroni correction, only ethanolamine (EA) levels remained significantly reduced in depressed patients (nominal *P* = 0.0000011). A substantial proportion of the depressed patients (40.5%) showed abnormally low CSF EA levels (<12.1 μM) (*P* = 0.000033; OR = 11.6, 95% CI: 3.1–43.2). When patients with low EA and those with high EA levels were compared, the former had higher scores for overall depression severity (*P* = 0.0033) and ‘Somatic Anxiety’ symptoms (*P* = 0.00026). In unmedicated subjects, CSF EA levels showed a significant positive correlation with levels of homovanillic acid (*P* = 0.0030) and 5-hydroxyindoleacetic acid (*P* = 0.019). To our knowledge, this is the first study showing that patients with MDD have significantly lower CSF EA concentrations compared with control subjects. CSF EA could be a state-dependent biomarker for a subtype of MDD.

Major depressive disorder (MDD) is a common disease with a prevalence rate estimated at 4.4% worldwide^1^. Since the pathophysiology of MDD remains elusive, no established biochemical marker is available for everyday use in the clinical setting and the diagnosis of MDD largely depends on the clinical interview^2^. Although many candidate molecules are present in peripheral blood^3^, no study has successfully found a biomarker that is of practical use in the diagnosis, subtyping, or symptomatic assessment of MDD.

Since cerebrospinal fluid (CSF) contacts the interstitial fluid in the central nervous system (CNS)^4^ and is mostly segregated from the peripheral circulation by the blood-brain barrier, CSF reflects molecular dynamics in the brain. The composition of CSF (electrolytes^5^, amino acids^6^, and proteins^7^) differs substantially from that of peripheral blood. Total tau and phosphorylated tau protein in CSF have been established as biomarkers for Alzheimer's disease^8^, but are not detectable in peripheral blood. It is therefore feasible to search for a biomarker for MDD in the CSF. The proteomics approach to CSF samples seems to be promising^9^.

We have focused on amino acids and related molecules in the CSF to identify a biomarker for MDD, because alterations in the serotonin, noradrenaline, dopamine, glutamate, and γ-amino-butyric acid (GABA) systems are implicated in MDD^10^. These neurotransmitters are themselves amino acids or are synthesized from amino acids. Previous studies examined amino acid levels in the peripheral blood of MDD patients, although their results are equivocal^11^,^12^,^13^,^14^. We have recently reported a meta-analysis demonstrating that the plasma L-tryptophan concentration is significantly lower in MDD patients than healthy controls (*P* = 0.000059)^15^. In this context, we chose to examine amino acids and related molecules in the CSF of MDD patients.

Although many studies compared CSF amino acid concentrations between MDD patients and healthy controls^16^,^17^,^18^,^19^,^20^,^21^,^22^,^23^,^24^,^25^,^26^,^27^,^28^, the majority examined a single amino acid or a few amino acids. Some researchers found reduced CSF GABA levels in depressed patients compared with controls^18^,^19^,^21^,^28^, while others reported contradictive negative results^17^,^20^,^22^. Increased glutamine levels in depressed patients^24^, and reduced glutamate and glycine concentrations in refractory patients were reported^25^, however, no differences in CSF glutamate and glutamine levels between 2 groups were inconsistently found^27^. No significant difference between patients and controls was found for tryptophan^21^,^23^, tyrosine^21^ or alanine^24^. To our knowledge, only two studies examined comprehensive amino acid profiles in CSF. An early study examined 32 amino acids and related molecules including ethanolamine (EA); however, that sample included only 8 subjects with unipolar depression and 2 controls^16^. A metabolomics-based approach in 14 currently-depressed patients, 14 remitted patients, and 18 controls found that CSF methionine levels were significantly increased in remitted patients compared with controls^26^. Thus, previous information on CSF amino acid levels in MDD patients is surprisingly limited.

We measured CSF amino acids and related molecules in a relatively large sample to search for a biomarker for MDD. We also analyzed the correlations between CSF amino acid levels and depression severity, psychotropic medication, and monoamine metabolites.

## Subjects and methods

### Subjects

Subjects were 52 patients with MDD and 54 healthy controls matched for age and sex. All participants were biologically-unrelated Japanese. Patients were recruited at the National Center of Neurology and Psychiatry (NCNP) Hospital (Tokyo, Japan), or through advertisements in free local magazines, and by our website. Healthy controls were from the same geographical area (i.e., western Tokyo metropolitan) via advertisements in magazines and website. Trained psychologists or psychiatrists conducted a structured Mini-International Neuropsychiatric Interview (M.I.N.I.)^29^, Japanese version with all participants. A consensus diagnosis was made according to the Diagnostic and Statistical Manual of Mental Disorders, 4^th^ edition (DSM-IV) criteria^30^ based on the M.I.N.I., an additional unstructured interview, and information from medical records if available. Patients with any comorbid axis I disorder were excluded, as were individuals with a prior medical history of CNS disease, severe head injury, or substance abuse/dependence. After the nature of the study procedures had been fully explained, written informed consent was obtained from all subjects. MDD symptoms were assessed using the Japanese version of the 17-item Hamilton Depression Rating Scale (HAMD-17)^31^, and the cut-off score for remission was ≤7^32^. Ten of the 52 patients were remitted. Among all MDD patients (depressed + remitted), 39 patients were medicated and the remaining 13 (11 depressed and 2 remitted) were not on psychotropic medication. Daily doses of benzodiazepine derivatives, antidepressants, and antipsychotics were converted to equivalent doses of diazepam, imipramine, and chlorpromazine respectively, using published guidelines^33^ When each class of drugs was not medicated, the dose was considered to be zero. The present experiments on our participants were conducted in accordance with the Declaration of Helsinki. The study protocol was approved by the ethics committee at the NCNP (No. 305).

### Sample collection

Between 10:00 h and 16:00 h, CSF samples were obtained by lumbar puncture from the L4–5 or L3–4 interspace of participants, in the left decubitus position. CSF samples were immediately placed on ice, and centrifuged at 4000 × *g*. Supernatants were aliquoted and stored at −80°C until assays were performed. All samples were collected from August 2010 to August 2013. Initial 2 mL of CSF was used for measuring glucose, chloride, total protein levels and monoamine metabolites. Homovanillic acid (HVA), 3-methoxy-4-hydroxyphenylethyleneglycol (MHPG), and 5-hydroxyindoleacetic acid (5-HIAA) were measured by high-performance liquid chromatography (HPLC) at SRL Co., Inc. (Tokyo, Japan).

### Determination of CSF levels of amino acids and related molecules by HPLC

CSF sample was mixed with 4% 5-sulfosalicylic acid dihydrate (WAKO, Tokyo, Japan), and centrifuged for 10 min at 12,000 × *g* and 4°C. Each supernatant was transferred to a micro-tube, filtered using a 0.22-μm pore-diameter syringe-filter (AS ONE, Osaka, Japan), and subjected to HPLC (JASCO, Tokyo, Japan). Acquired data were processed and quantified on the chromatography data station ChromNAV (JASCO). A detailed description of the HPLC protocol is available in the Supplementary Methods.

### Validation by capillary electrophoresis time-of-flight mass spectrometry (CE-TOF-MS)

Amino acids and related molecules were also measured using CE-TOF-MS for a subset of the subjects (24 depressed, 3 remitted patients, and 27 controls). The CE-TOF-MS measurements were performed at Human Metabolome Technologies, Inc. (Yamagata, Japan), by coauthors who were blinded to the HPLC data obtained at the NCNP. Compounds were identified by their peaks using annotated tables with *m*/*z* values and normalized by migration times. Detailed CE-TOF-MS procedures are described elsewhere^34^.

### Statistical analysis

Data are reported as means ± standard deviation (*SD*). Means were compared using *t*-tests or analysis of variance. Categorical variables were compared using the *χ^2^* test with exact probability. Analysis of covariance (ANCOVA), controlling for age and sex, was performed to compare CSF levels of amino acids and related molecules in the patients and controls and between subgroups of the patients. Partial correlation analysis, controlling for age and sex, was performed to examine the correlations between equivalent doses of psychotropic drugs and concentrations of amino acids and related molecules, and between EA levels and other CSF substances including biogenic amine metabolites. The Mann-Whitney *U* test with exact probability was used to compare clinical symptoms (HAMD-17 scores) between MDD subgroups (Low-EA vs. High-EA). We obtained 95% CI for the difference between two medians for Mann-Whitney *U* using Hodges-Lehmann estimate. HAMD-17 items were assigned to the following subscales: ‘Core’ (items 1, 2, 7, 8, 10, 13), ‘Sleep’ (items 4, 5, 6), ‘Activity’ (items 7, 8), ‘Psychic Anxiety’ (items 9, 10), and ‘Somatic Anxiety’ (items 11, 12, 13), according to Serretti *et al.*^35^. We used non-parametric estimate of receiver operating characteristic (ROC) curves for the assessment of specificity and sensitivity of EA to discriminate between MDD patients and controls, and between depressed and remitted MDD groups. Statistical significance for a two-tailed *P*-value was <0.05, and was corrected for multiple comparisons using the Bonferroni method. All analyses were performed using IBM SPSS Statistics 22.0 Japanese version (IBM Japan, Tokyo, Japan).

## Results

Demographic and clinical characteristics of the subjects are shown in Table 1. There were no significant differences in sex ratio, age distribution, time of CSF sampling, or number or days from sample collection among the depressed, remitted, and control subjects. No significant difference was observed in psychotropic drug doses between depressed and remitted MDD groups. CSF concentrations of 25 of the 41 amino acids and related molecules were successfully determined in the majority of subjects (Table 2). There was nominally a significant difference in the concentrations of threonine, glutamine, EA, and carnosine between the depressed MDD and control groups. When the critical *P* = 0.002 (0.05/25) was conservatively applied after the Bonferroni correction, only EA remained significant (nominal *P* = 0.0000011).

**Figure 1:**
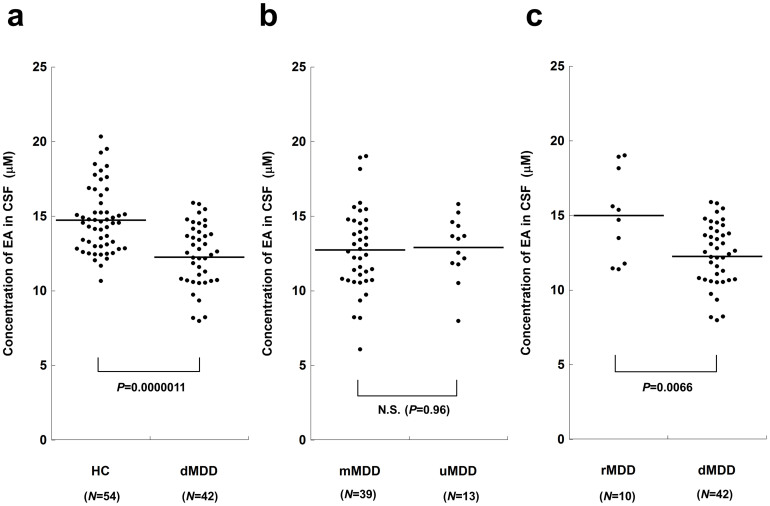
Dot plots of CSF EA concentrations in patients with MDD and controls. Horizontal bars represent mean values of the groups. Statistical analyses were performed by ANCOVA, controlling for age and sex. (a) Comparison between dMDD patients and HC, (b) that between mMDD and uMDD, and (c) that between rMDD and dMDD. Abbreviations: CSF, cerebrospinal fluid; EA, ethanolamine; HC, healthy controls; dMDD, depressed (non-remitted) patients with major depressive disorder; rMDD, remitted patients; mMDD, medicated patients; uMDD, unmedicated patients; ANCOVA, analysis of covariance.

We then performed detailed analyses on the possible relationships between CSF EA levels and clinical variables. In controls, there was no significant difference in CSF EA levels between men and women controlled for age (*F* = 0.045, *df* = 1, *P* = 0.83; 95% CI: −1.10 to 1.36), and no significant correlation between EA and age controlled for sex (*r* = −0.063, *df* = 51, *P* = 0.66; 95% CI: −0.33 to 0.21), between EA and time of CSF sampling controlled for sex and age (*r* = 0.061, *df* = 50, *P* = 0.67: 95% CI: −0.22 to 0.33), or between EA and number of days from sample collection controlled for sex and age (*r* = 0.0062, *df* = 50, *P* = 0.97; 95% CI: −0.27 to 0.28), in a partial correlation analysis. Figure 1a shows dot plots of CSF EA concentrations in depressed MDD patients and controls. Among all MDD patients, no significant difference in EA levels was observed between the medicated and unmedicated patients using ANCOVA, controlling for sex and age (*F* = 0.0030, *df* = 1, *P* = 0.96; 95%CI: −1.57 to 1.66) (Figure 1b), and there were no significant differences in age, sex, or total HAMD-17 score. When unmedicated MDD patients and controls were compared, there was a significant difference in CSF EA based on ANCOVA (*F* = 7.0, *df* = 1, *P* = 0.010; 95% CI: −3.28 to −0.46). Remitted MDD patients had significantly higher EA levels than depressed MDD patients (ANCOVA; *F* = 8.1, *df* = 1, *P* = 0.0066; 95%CI: 0.67 to 3.90) (Figure 1c), and they showed no significant differences in age or sex. There was no significant difference in CSF EA levels between remitted patients and controls (*F* = 0.0092, *df* = 1, *P* = 0.92; 95%CI: −1.68 to 1.53). These comparisons for CSF EA concentrations between subgroups are summarized in Table 3. Area under curve (AUC) by the ROC curve to discriminate between depressed (non-remitted) MDD patients and controls was 0.77 (Supplementary Fig. S1a), that between depressed and remitted MDD groups was 0.75 (Supplementary Fig. S1b), indicating ‘fair test’ for these discriminations. When “abnormally low CSF EA levels” were defined as the 5^th^ percentile value of the controls (<12.1 μM), 17 depressed patients (40.5%) and 3 controls fell within this range (*χ^2^* = 17.5, *df* = 1, *P* = 0.000033; OR = 11.6, 95% CI: 3.1 to 43.2).

**Figure 2:**
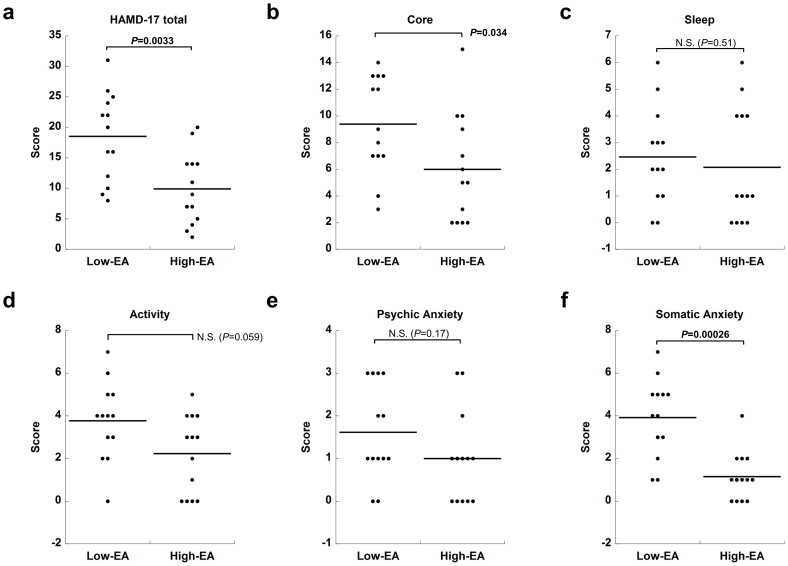
Dot plots of HAMD-17 total score and subscale scores in High and Low-EA patients. We defined MDD patients with CSF EA values below the 1^st^ quartile as ‘Low-EA’ (*N* = 13), and MDD patients with CSF EA values above the 3^rd^ quartile as ‘High-EA’ (*N* = 13) to compare the CSF EA levels with symptoms. Horizontal bars in dot clusters represent mean values of the groups. *P*-values were obtained by the Mann-Whitney *U* test. (a) Low-EA showed a significantly higher total score for HAMD-17 than High-EA (*U* = 29.0, *P* = 0.0033; 95% CI: 3.0 to 15.0). (b) Low-EA showed a significantly higher score for the ‘Core’ subscale than High-EA (*U* = 43.5, *P* = 0.034; 95% CI: 1.0 to 7.0). There were no significant differences between the two groups for (c) ‘Sleep’ (*U* = 71.5, *P* = 0.51; 95% CI: −1.0 to 2.0), (d) ‘Activity’ (*U* = 48.0, *P* = 0.059; 95% CI: 0.0 to 3.0), and (e) ‘Psychic Anxiety’ (*U* = 57.5, *P* = 0.17; 95% CI: 0.0 to 2.0). (f) Low-EA showed a significantly higher score for ‘Somatic Anxiety’ than High-EA (*U* = 18.5, *P* = 0.00026; 95% CI: 1.0 to 4.0). Abbreviations: HAMD-17, 17-item Hamilton Depression Rating Scale; CSF, cerebrospinal fluid; EA, ethanolamine; Low-EA, patients with CSF EA values below the 1^st^ quartile; High-EA, patients with CSF EA values above the 3^rd^ quartile; CI, confidence interval for the difference between two medians.

To investigate the relationship between patient's symptoms and CSF EA levels, we defined MDD patients (depressed + remitted) whose levels fell below the 1^st^ quartile of CSF EA values as ‘Low-EA’ (*N* = 13) and above the 3^rd^ quartile as ‘High-EA’ (*N* = 13). The Low-EA group had higher HAMD-17 total scores (*P* = 0.0033, Mann-Whitney *U* test), and ‘Core’ (*P* = 0.034) and ‘Somatic Anxiety’ (*P* = 0.00026) subscale scores (Figure 2). Other HAMD subscales (‘Sleep’, ‘Activity’, and ‘Psychic Anxiety’) showed no significant differences between Low-EA and High-EA groups.

Although there was no significant difference in CSF EA levels between medicated and unmedicated MDD patients, we examined the possible correlation between the equivalent dose of psychotropic drugs and CSF amino acid levels in the depressed MDD group (Table S1). CSF EA levels did not significantly correlate with benzodiazepine derivatives (*P* = 0.29), antidepressants (*P* = 0.34), or antipsychotics (*P* = 0.21). With respect to other molecules, several amino acid concentrations nominally showed significant correlations with medications (Supplementary Table S1). The correlations between isoleucine and antidepressants (*P* = 0.00056) (Supplementary Fig. S2), remained significant even after correcting for multiple comparisons [critical *P*-value of 0.05/(25 × 2) = 0.001].

**Figure 3:**
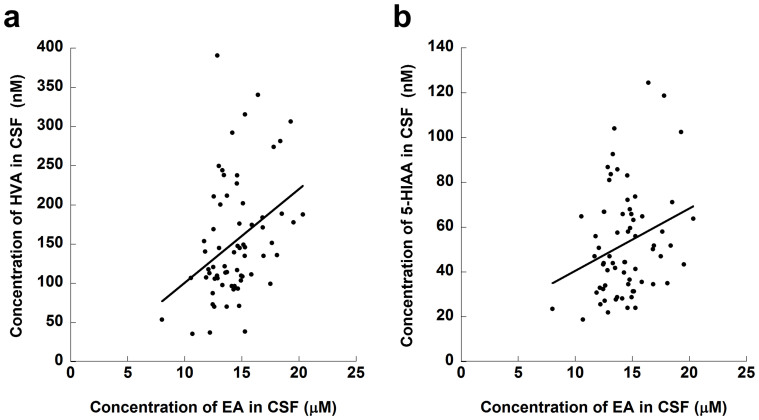
Scatter plots and regression lines for the relationships of EA with HVA and 5-HIAA in CSF. Partial correlation test controlling for age and sex was performed in healthy controls (*N* = 54) and unmedicated patients with major depressive disorder (*N* = 13). Significant correlations of EA with (a) HVA and (b) 5-HIAA in CSF are shown. Abbreviations: EA, ethanolamine; CSF, cerebrospinal fluid; HVA, homovanillic acid; 5-HIAA, 5-hydroxyindoleacetic acid.

We further investigated the correlations between CSF EA levels and other CSF substances in unmedicated patients with MDD and controls (total *N* = 67), based on reports that levels of CSF biogenic amine metabolites are affected by psychotropic drugs^36^. HVA, a catabolite of dopamine (*P* = 0.0030), and 5-HIAA, a catabolite of serotonin (*P* = 0.019), showed significant correlations, while other substances (total protein, glucose, chloride, and MHPG) did not (Figure 3 and Table 4).

To validate the HPLC measurements, CSF EA levels were measured in a subset of the subjects using CE-TOF-MS. The EA values obtained using CE-TOF-MS and HPLC methods showed a near-perfect correlation (*r* = 0.89, *P* < 5 × 10^−18^) in partial correlation analysis, with age and sex as covariates (Supplementary Fig. S3a). Similar to Figure 1a, a significant difference in CSF EA values based on CE-TOF-MS data was observed between the depressed MDD patients and controls (*P* = 0.0052, ANCOVA) (Supplementary Fig. S3b).

## Discussion

Several of our findings are potentially of clinical significance. The levels of some CSF amino acids differed between MDD patients and controls. EA (also known as monoethanolamine or 2-aminoethanol), in particular, remained significantly lower in patients than controls after correcting for multiple comparisons (*P* = 0.0000011). Notably, as many as 40% of the depressed patients showed abnormally low levels, and EA levels were significantly lower in depressed patients than remitted patients. When relationships with clinical variables were examined in the patients, the Low-EA group showed higher HAMD-17 and subscale scores compared with the High-EA group. CSF EA did not correlate with the dose of psychotropic drugs, although isoleucine showed a correlation with antidepressants even after correcting for multiple testing. Lastly, CSF EA values were significantly correlated with CSF HVA and 5-HIAA levels in unmedicated subjects.

An early study by Goodnick *et al*.^16^ reported that CSF tyrosine levels differed across diagnostic groups (bipolar, unipolar depression, and controls). The authors reported the CSF EA levels; however, there were only two controls in their sample and it is difficult to compare their results with ours. Frye *et al.*^25^ reported that CSF glutamate and glycine levels were decreased in patients with mood disorder. By contrast, we found no significant difference in the levels of either amino acid between depressed patients and controls. One of the reasons for the inconsistency may be that the majority of the patients in Frye *et al*.'s study had bipolar disorder. Another reason may be that their subjects were all unmedicated; however, we could not confirm the results of Frye *et al*. even when our unmedicated patients were compared with controls (data not shown). Levine *et al*. reported that depressed patients (MDD and bipolar disorder) showed increased CSF glutamine levels than controls^24^. CSF glutamine levels in our MDD patients were also significantly increased than in controls. However, there was no significant difference between our unmedicated patients (*N* = 11) and controls (629.5 ± 72.9 vs. 644.3 ± 108.8 μM, *F* = 0.046, *df* = 1, *P* = 0.83, 95% CI: −69.33 to 55.89). Therefore, the observed increase in glutamine in our total subjects may be attributable to medication. Regarding CSF GABA, several previous reports^18^,^19^,^21^,^28^ showed its decrease in depressed subjects; however, we observed no significant differences between patients and controls, which is in line with other studies^17^,^20^,^22^. Recently, Kaddurah-Daouk *et al*., who employed a metabolomics-based approach, reported that methionine was increased in remitted MDD patients compared with depressed patients and healthy controls^26^. Our subjects showed similar results. Although CSF methionine levels did not differ significantly between the currently depressed patients and controls, our remitted patients had significantly higher CSF methionine levels than depressed patients (4.1 ± 1.2 vs. 3.3 ± 0.9 μM, *F* = 9.1, *df* = 1, *P* = 0.0041; 95% CI: 0.31 to 1.57, ANCOVA) and controls (vs. 3.5 ± 0.8, *F* = 5.7, *df* = 1, *P* = 0.020; 95% CI: 0.11 to 1.29). Methionine might be involved in the recovery processes of MDD and could be a biomarker for remission.

The significantly higher levels of CSF EA in remitted patients compared with depressed patients suggest that CSF EA levels might be state-dependent. In line, our Low-EA patients showed a higher HAMD-17 total score than the High-EA group. Vagus nerve stimulation (VNS), which is effective for MDD patients and alters the metabolites of neurotransmitters^37^, was reported to elevate CSF EA levels in epileptic patients^38^. VNS may exert its effect through mechanisms that increase central EA. Longitudinal studies are warranted to examine whether antidepressant treatments increase CSF EA.

When we defined abnormally low EA levels based on the 5^th^ percentile of the controls, approximately 40% of the depressed patients fell into this range, suggesting that a substantial proportion of subjects with MDD could be distinguished from normal subjects based on CSF EA levels, which would be useful for diagnosis. MDD patients with low CSF EA levels may constitute a subtype of MDD. Indeed, the Low-EA group was characterized by higher ‘Core’ and ‘Somatic Anxiety’ symptoms. In addition, we found a significant positive correlation between CSF EA and HVA and 5-HIAA levels, suggesting that MDD characterized by low CSF EA levels reflects impaired dopaminergic and serotonergic functions in the CNS, possibly due to synaptic dysregulation by an altered endocannabinoid system (see below).

Since most of our patients were medicated, the observed decrease in CSF EA may be attributable to medication. However, this possibility is unlikely because CSF EA was decreased in unmedicated patients compared with controls; there was no significant difference in CSF EA levels between medicated and unmedicated patients; and there was no significant correlation between CSF EA levels and the dose of any class of psychotropic drugs. Our results therefore suggest that CSF EA could be a useful biomarker even in medicated patients. With respect to the effect of antidepressants, we found a significant correlation with CSF isoleucine levels even after correcting for multiple comparisons. To our knowledge, no study has examined the effect of antidepressants on CSF amino acid levels; therefore, further studies are warranted.

EA is closely related to endocannabinoid signaling in the CNS (see Supplementary Fig. S4). EA is both a precursor to, and a metabolite of, anandamide (*N*-arachidonoylethanolamine), a ligand for cannabinoid receptors (CBs), and transient receptor potential vanilloid type 1 (TRPV1). The endocannabinoid system is implicated in depression, suicide, and stress-related affective disorders^39^. CB_1_ receptor density is high at presynaptic axon terminals, where it functions to inhibit neurotransmitter release^40^. This may substantiate our observation that CSF EA was correlated with HVA and 5-HIAA levels. In human studies, inconsistent results have been reported on serum endocannabinoid levels in MDD^41^,^42^,^43^. One study found no significant difference in the CSF anandamide level between MDD patients and controls, although it was elevated in unmedicated patients with schizophrenia^44^. Anandamide is synthesized on demand, binds with high affinity to extracellular CB_1_ receptors, and is rapidly inactivated by active transport into neurons, followed by hydrolysis^45^. EA might be a stable surrogate marker for the anandamide system.

Decreased CSF EA may be due to inflammatory responses that have been implicated in MDD^46^. We previously reported elevated CSF IL-6 levels in MDD^47^ suggesting the involvement of neuroinflammation. In the inflammatory process, activation of microglia and upregulation of cyclooxygenase-2 may facilitate conversion of EA to *N*-acylethanolamines or prostaglandin H_2_ ethanolamide^48^,^49^.

There are several limitations to this study. Firstly, the numbers of unmedicated patients with MDD (*N* = 13) and remitted individuals (*N* = 10) were small. However, we detected a significant difference in CSF EA levels between unmedicated patients and controls; and between depressed and remitted patients, which suggests large effect sizes. Secondly, the measurement of the CSF sample took place in a real-world setting; the majority of patients were medicated, and sampling was not performed after fasting or at a fixed time. However, we did not observe any correlation of EA with psychotropic medication or CSF sampling time. This makes CSF EA a feasible biomarker for everyday use in the clinical setting. Nevertheless, studies are necessary to elucidate the possible effects of fasting. Thirdly, there were missing values for several amino acids (see Table 2), which were likely due to small values below the detection limit and might have caused false negative results. Fourthly, small proportion of patients (*N* = 13) received antipsychotic medication, which may have an effect on CSF EA levels. However, there was no significant correlation between daily chlorpromazine equivalent doses of antipsychotics and CSF EA levels; therefore, the possible effect might be minimal. Finally, we obtained data only for MDD patients and controls. Further studies on other neuropsychiatric disorders are necessary to determine whether low EA is specific to MDD.

In conclusion, we found, for the first time, that CSF EA levels were reduced independently of medication in a substantial proportion (40%) of depressed MDD patients. Such patients had characteristic symptomatology (i.e., ‘Somatic Anxiety’) and CSF monoamine metabolite profiles (i.e., reduced HVA and 5-HIAA), and thus constitute a subtype of MDD.

## Author Contributions

S.O. designed the study, managed the literature searches, profiled the CSF samples by HPLC, undertook the statistical analyses, and wrote the draft of the manuscript. K.H. and Y.Y. recruited the participants. K.H. and D.S. diagnosed the participants, and collected the CSF samples. K.H. selected the sample set. Y.Y. and R.M. made psychological assessments. R.M. created and maintained database system. M.O., H.H. and T.T. screened the participants and diagnosed the patients. Y.O. and H.S. performed the CE-TOF-MS measuring for data validation. N.M. and J.M. reviewed the draft and gave critical comments on the manuscript. T.H., S.Y. and T.N. contributed to the recruitment of clinical volunteers. H.K. supervised the entire project and gave critical comments on the manuscript. All authors contributed to and have approved the final manuscript.

## Supplementary Material

Supplementary Information Supplementary Materials

## Figures and Tables

**Table 1:** Demographic and Clinical data on subjects

	dMDD	rMDD	HC	Statistics
***N* (Male/Female)**	42 (19/23)	10 (6/4)	54 (28/26)	*χ*^2^ = 0.86, *df* = 2, *P* = 0.63 1
**Age, year**	45.5 ± 12.2	42.9 ± 14.9	43.6 ± 15.3	*F* = 0.26, *df* = 2, *P* = 0.77 2
**BZD, mg/day (ratio)**	14.1 ± 14.4 (29/42)	12.1 ± 17.9 (7/10)	N.A.	*t* = 0.38, *df* = 50, *P* = 0.71, 95% CI: −8.66 to 12.63 3
**AD, mg/day (ratio)**	136.9 ± 131.1 (27/42)	149.4 ± 170.4 (7/10)	N.A.	*t* = −0.26, *df* = 50, *P* = 0.80, 95% CI: −110.72 to 85.78 3
**AP, mg/day (ratio)**	83.7 ± 176.2 (13/42)	56.5 ± 125.5 (3/10)	N.A.	*t* = 0.46, *df* = 50, *P* = 0.65, 95% CI: −91.67 to 146.14 3
**HAMD-17, score**	18.0 ± 7.2	4.3 ± 2.4	N.A.	*t* = 10.20, *df* = 44.93, *P* < 5 × 10^−13^, 95% CI: 9.00 to 18.36 3
**Time of CSF sampling, min **4	185.2 ± 95.4	221.5 ± 111.9	172.2 ± 118.6	*F* = 0.89, *df* = 2, *P* = 0.42 2
**Days from CSF sampling, day **5	554.9 ± 313.5	721.5 ± 276.9	479.7 ± 286.4	*F* = 3.01, *df* = 2, *P* = 0.054 2

^1^Based on the chi-squared test with exact probability.

^2^Based on the analysis of variance.

^3^Based on the *t*-test.

^4^Sampling time is expressed as minutes from 10:00 AM.

^5^Number of days from the lumbar puncture day to Sep 1, 2013.

Abbreviations: dMDD, depressed (non-remitted) patients with major depressive disorder; rMDD, remitted patients; HC, healthy controls; HAMD-17, 17-item Hamilton Depression Rating Scale; CSF, cerebrospinal fluid; BZD, daily diazepam equivalent dose of benzodiazepine derivatives; AD, daily imipramine equivalent dose of antidepressants; AP, daily chrolpromazine equivalent dose of antipsychotics; CI, confidence interval for the difference between two means.

**Table 2:** Comparisons between dMDD and HC for amino acids and related molecule profiles in cerebrospinal fluid

Name	dMDD (*N* = 42)		HC (*N* = 54)		Statistics for ANCOVA			
	μM ± *SD*	NMISS 1	μM ± *SD*	NMISS	*F*	*df*	*P*	95% CI
**Phosphoethanolamine**	4.7 ± 1.2	0	5.1 ± 1.1	0	2.12	1	0.15	−0.75 to 0.11
**Threonine**	33.1 ± 9.3	0	29.8 ± 5.3	0	4.30	1	0.041	0.13 to 6.030
**Serine**	29.3 ± 7.5	0	27.9 ± 6.1	0	0.75	1	0.39	−1.51 to 3.86
**Asparagine**	7.2 ± 1.4	0	6.6 ± 1.3	0	3.85	1	0.053	−0.0065 to 1.11
**Glutamine**	711.3 ± 142.5	0	644.3 ± 108.8	0	6.70	1	0.011	15.16 to 115.15
**Glycine**	6.4 ± 2.0	0	6.0 ± 1.8	0	0.95	1	0.33	−0.38 to 1.12
**Alanine**	37.5 ± 10.3	0	33.7 ± 9.0	0	3.34	1	0.071	−0.31 to 7.47
**α-Amino-n-butyric acid**	2.7 ± 1.0	0	2.6 ± 0.9	0	0.30	1	0.59	−0.29 to 0.51
**Valine**	16.6 ± 4.8	0	15.2 ± 4.6	0	2.41	1	0.12	−0.39 to 3.23
**Methionine**	3.3 ± 0.9	0	3.5 ± 0.8	0	1.68	1	0.20	−0.56 to 0.12
**Isoleucine**	5.2 ± 1.5	0	4.8 ± 1.4	0	2.88	1	0.093	−0.080 to 1.017
**Leucine**	12.5 ± 3.4	0	11.5 ± 2.9	0	3.42	1	0.068	−0.082 to 2.27
**Tyrosine**	9.0 ± 1.8	0	9.4 ± 2.3	0	1.50	1	0.22	−1.34 to 0.32
**Phenylalanine**	10.1 ± 2.1	0	10.0 ± 2.3	0	0.038	1	0.85	−0.79 to 0.96
**Ethanolamine**	12.3 ± 2.3	0	14.8 ± 2.2	0	**27.36**	**1**	**0.0000011 **2	**−3.29 to −1.48**
**Lysine**	24.3 ± 7.1	0	22.2 ± 3.9	0	3.11	1	0.081	−0.25 to 4.17
**Histidine + 1-Methylhistidine**	8.4 ± 2.7	0	8.0 ± 1.4	0	0.69	1	0.41	−0.50 to 1.21
**Arginine**	21.8 ± 5.1	0	22.6 ± 4.7	0	0.58	1	0.45	−2.79 to 1.25
**Aspartate**	0.7 ± 0.3	17	0.7 ± 0.2	21	1.091	1	0.30	−0.062 to 0.20
**Glutamate**	9.1 ± 5.4	2	8.3 ± 5.2	1	0.57	1	0.45	−1.39 to 3.088
**Cystine**	2.9 ± 0.7	6	2.8 ± 0.8	8	0.20	1	0.66	−0.25 to 0.40
**Tryptophan**	1.8 ± 0.7	19	2.0 ± 0.4	18	2.64	1	0.11	−0.55 to 0.058
**Ornithine**	2.9 ± 1.4	6	3.2 ± 1.5	7	1.52	1	0.22	−1.034 to 0.24
**Carnosine**	2.4 ± 1.5	7	3.3 ± 2.1	10	5.30	1	0.024	−1.58 to −0.11
**γ-Aminobutyric acid**	0.3 ± 0.1	24	0.3 ± 0.1	30	1.061	1	0.31	−0.023 to 0.072

^1^Missing values are replaced with blanks in the ANCOVA analysis.

^2^Adjusted significance was set at *P* < 0.002, and significant *P*-values after Bonferroni correction are in bold type.

Abbreviations: dMDD, depressed (non-remitted) patients with major depressive disorder; HC, healthy controls; SD, standard deviation; NMISS, number of missing values; ANCOVA, analysis of covariance; CI, confidence interval for the difference between two means.

**Table 3:** Summarized comparisons in CSF EA concentrations between subgroups

Comparisons		Differences		Statistics for ANCOVA 1	95% CI
**dMDD (*N* = 42)** **vs HC (*N* = 54)**		12.3 ± 2.3 vs 14.8 ± 2.2		***F* = 27.36, *df* = 1, *P* = 0.0000011**	**−3.29 to −1.48**
**mMDD (*N* = 39) vs uMDD (*N* = 13)**		12.8 ± 2.8 vs 12.9 ± 2.1		*F* = 0.0030, *df* = 1, *P* = 0.96	−1.57 to 1.66
**dMDD (*N* = 42)** **vs rMDD (*N* = 10)**		12.3 ± 2.3 vs 15.0 ± 3.0		***F* = 8.073, *df* = 1, *P* = 0.0066**	**−3.90 to −0.67**

^1^Statistical values derived from ANCOVA controlling for sex and age.

Significant *P*-values are shown in bold type.

Abbreviations: CSF, cerebrospinal fluid; EA, ethanolamine; ANCOVA, analysis of covariance; dMDD, depressed (non-remitted) patients with major depressive disorder; rMDD, remitted patients; HC, healthy controls; uMDD, unmedicated patients; mMDD, medicated patients; CI, confidence interval for the difference between two means.

**Table 4:** Partial correlations between levels of CSF substances and EA concentrations

Substances	Statistics			
	*r*	*df*	*P*	95% CI
**Total protein**	−0.055	63	0.66	−0.30 to 0.19
**Glucose**	−0.075	63	0.55	−0.31 to 0.17
**Chloride**	0.077	63	0.54	−0.17 to 0.31
**HVA**	0.36	63	**0.0030 **1	**0.13 to 0.56**
**MHPG**	0.14	63	0.27	−0.11 to 0.37
**5-HIAA**	0.29	63	**0.019**	**0.051 to 0.50**

^1^Significant *P*-values in bold type.

Abbreviations: CSF, cerebrospinal fluid; EA, ethanolamine; CI, confidence interval; HVA, homovanillic acid; MHPG, 3-methoxy-4-hydroxyphenylethyleneglycol; 5-HIAA, 5-hydroxyindoleacetic acid.
