# Supplementary material for: Reduced cerebrospinal fluid ethanolamine concentration in major depressive disorder
Source: Sci Rep. 2015 Jan 15;5:7796. doi: 10.1038/srep07796 (PMC4295090; doi:10.1038/srep07796)
Supplement: Supplementary Information — Supplementary Materials [file srep07796-s1.doc]

**Reduced cerebrospinal fluid ethanolamine concentration
 in major depressive disorder**

Shintaro Ogawa ^1, 7^, Kotaro Hattori ^1, 2^, Daimei Sasayama ^1, 3^, Yuki Yokota ^1^, ^2^, Ryo Matsumura ^1^, ^2^, Junko Matsuo ^1, 7^, Miho Ota ^1^, Hiroaki Hori ^1^, Toshiya Teraishi ^1^, Sumiko Yoshida ^1^, Takamasa Noda ^4^, Yoshiaki Ohashi ^5^, Hajime Sato ^5^, Teruhiko Higuchi ^6^, Nobutaka Motohashi ^7^, Hiroshi Kunugi ^1^

^1^ Department of Mental Disorder Research, National Institute of Neuroscience, National Center of Neurology and Psychiatry, Kodaira, Tokyo, 187-8502, Japan

^2^ Translational Medical Center, National Center of Neurology and Psychiatry, Kodaira, Tokyo, 187-8551, Japan

^3^ Department of Psychiatry, Shinshu University School of Medicine, Matsumoto, Nagano, 390-8621, Japan

^4^ Department of Psychiatry, National Center Hospital, National Center of Neurology and Psychiatry, Kodaira, Tokyo, 187-8551, Japan

^5^ Human Metabolome Technologies, Inc., Tsuruoka, Yamagata, 997-0052, Japan

^6^ National Center of Neurology and Psychiatry, Kodaira, Tokyo, 187-8551, Japan

^7^ Department of Neuropsychiatry, Interdisciplinary Graduate School of Medicine and Engineering, University of Yamanashi, Chuo, Yamanashi, 409-3898, Japan

**Supplemental Methods**

*Determination of levels of amino acids and related molecules in the cerebrospinal fluid (CSF)*

A 60-μL volume of CSF sample was mixed with 60 μL of 4% 5-sulfosalicylic acid dihydrate (WAKO, Tokyo, Japan), and the final concentration of 5-sulfosalicylic acid dihydrate was 2%. After standing for 30 min, the mixture was centrifuged for 10 min at 12,000 × *g* and 4°C. The supernatant was transferred into a micro-tube, filtered using 0.22-μm pore-diameter syringe-filter (AS ONE, Osaka, Japan). The sample was analyzed using an automated high-performance liquid chromatography (HPLC) system (JASCO, Tokyo, Japan) for measuring CSF amino acids and related molecule concentrations. We prepared 41 standards for the absolute calibration curve using the Amino Acids Mixture Standard Solution, Type B and Type AN-2 (WAKO) and an additional standard solution mixture for tryptophan, glutamine, theanine, proline, 2,6-diaminopimeric acid, and asparagine (all purchased from WAKO). Acquired data were processed and the 41 amino acids and related molecules were quantified using the Chromatography data station ChromNAV (JASCO).

The HPLC system consisted of a quaternary low-pressure gradient pump (PU-2089 plus), two semi-micro pumps (PU-2085 plus), an auto-sampler (AS-2057 plus), a column oven (CO-2065 plus), and a fluorescence detector (FP-2025 plus).

We used four buffers and two reagents, which are commercially available for the LC-2000 Li-LG amino acid measurement system. The first buffer contains lithium citrate tetrahydrate, ethanol, perchloric acid, and H_2_O. The second buffer contains lithium citrate tetrahydrate, thiodiglycol, perchrolic acid, and H_2_O. The third buffer contains lithium citrate tetrahydrate, lithium chloride, thiodiglycol, perchrolic acid, benzyl alcohol, ethanol, and H_2_O. The fourth buffer contains lithium hydroxide and H_2_O. The Hypo reagent, which is used to facilitate the reaction of proline and hydroxyproline with ortho-phthalaldehyde (OPA) by opening the ring, contains potassium hydroxide, orthoboric acid, sodium hypochlorite solution, and H_2_O. The OPA reagent contains potassium hydroxide, orthoboric acid, Brij-35 (polyoxyethylene lauryl ether), 3-mercaptopropionic acid, ethanol, ortho-phthalaldehyde, and H_2_O.

We set the control program of the quaternary gradient pump according to the manufacturer’s instruction. The flow rates of the four buffers and two reagents were set at 350 and 200 μL/min, respectively. The prepared samples were separated on a strong cation exchange column (AApack Li-LG, 5.0 μm, 6.0 mm ID × 80 mm, JASCO) in the HPLC system, and the temperature of the column oven was maintained at 38 °C. The samples were subsequently mixed with Hypo and OPA reagents for post-column derivatization of amino group and labeling with OPA. The fluorescence levels of the samples were measured using a fluorescent detector, with the wavelengths set at 345 nm (excitation) and 455 nm (emission).

| **Supplemental Table 1.** Partial correlations between daily equivalent doses of psychotropic drugs and concentrations of CSF amino acid and related molecules in the dMDD group | | | | | | | | | | | |
| --- | --- | --- | --- | --- | --- | --- | --- | --- | --- | --- | --- |
| **Name** |  | **BZD, diazepam equivalent dose** | | | |  | **AD, imipramine equivalent dose** | | | |  |
|  |  | ***r*** | ***df*** | ***P*** | **95% CI** |  | ***r*** | ***df*** | ***P*** | **95% CI** |  |
| **Phosphoethanolamine** |  | 0.24 | 38 | 0.14 | -0.080 to 0.51 |  | 0.18 | 38 | 0.27 | -0.14 to 0.46 |  |
| **Aspartate** |  | 0.28 | 21 | 0.19 | -0.15 to 0.62 |  | -0.037 | 21 | 0.87 | -0.44 to 0.38 |  |
| **Threonine** |  | 0.23 | 38 | 0.16 | -0.093 to 0.50 |  | 0.26 | 38 | 0.11 | -0.058 to 0.53 |  |
| **Serine** |  | 0.047 | 38 | 0.77 | -0.27 to 0.35 |  | -0.00044 | 38 | 1.00 | -0.31 to 0.31 |  |
| **Asparagine** |  | 0.082 | 38 | 0.61 | -0.24 to 0.38 |  | 0.13 | 38 | 0.42 | -0.19 to 0.42 |  |
| **Glutamate** |  | -0.27 | 36 | 0.097 | -0.55 to 0.051 |  | -0.08 | 36 | 0.63 | -0.39 to 0.25 |  |
| **Glutamine** |  | 0.29 | 38 | 0.065 | -0.019 to 0.55 |  | -0.0023 | 38 | 0.99 | -0.31 to 0.31 |  |
| **Glycine** |  | -0.15 | 38 | 0.34 | -0.44 to 0.17 |  | -0.012 | 38 | 0.94 | -0.32 to 0.30 |  |
| **Alanine** |  | 0.17 | 38 | 0.28 | -0.15 to 0.46 |  | 0.30 | 38 | 0.064 | -0.018 to 0.56 |  |
| **α-Amino-n-butyric acid** |  | 0.18 | 38 | 0.28 | -0.14 to 0.46 |  | 0.15 | 38 | 0.35 | -0.17 to 0.44 |  |
| **Valine** |  | 0.25 | 38 | 0.12 | -0.064 to 0.52 |  | 0.35 | 38 | **0.027** | **0.043 to 0.60** |  |
| **Cystine** |  | 0.17 | 32 | 0.35 | -0.18 to 0.48 |  | -0.19 | 32 | 0.29 | -0.49 to 0.16 |  |
| **Methionine** |  | 0.29 | 38 | 0.072 | -0.027 to 0.55 |  | 0.34 | 38 | **0.031** | **0.034 to 0.59** |  |
| **Isoleucine** |  | 0.32 | 38 | **0.045** | **0.0087 to 0.57** |  | 0.52 | 38 | **0.00056** | **0.25 to 0.72** |  |
| **Leucine** |  | 0.35 | 38 | **0.025** | **0.048 to 0.60** |  | 0.47 | 38 | **0.0021** | **0.19 to 0.68** |  |
| **Tyrosine** |  | 0.17 | 38 | 0.30 | -0.15 to 0.45 |  | 0.46 | 38 | **0.0030** | **0.17 to 0.67** |  |
| **Phenylalanine** |  | 0.1 | 38 | 0.53 | -0.22 to 0.40 |  | 0.22 | 38 | 0.17 | -0.095 to 0.50 |  |
| **Tryptophan** |  | 0.40 | 19 | 0.074 | -0.040 to 0.71 |  | 0.18 | 19 | 0.43 | -0.27 to 0.57 |  |
| **Ethanolamine** |  | -0.17 | 38 | 0.29 | -0.46 to 0.15 |  | 0.16 | 38 | 0.34 | -0.16 to 0.45 |  |
| **Ornithine** |  | 0.13 | 32 | 0.47 | -0.22 to 0.45 |  | -0.0023 | 32 | 1.00 | -0.34 to 0.34 |  |
| **Lysine** |  | 0.16 | 38 | 0.31 | -0.16 to 0.45 |  | 0.38 | 38 | **0.016** | **0.076 to 0.62** |  |
| **Histidine+1-Methylhistidine** |  | 0.13 | 38 | 0.43 | -0.19 to 0.42 |  | -0.11 | 38 | 0.51 | -0.40 to 0.21 |  |
| **Carnosine** |  | -0.095 | 31 | 0.60 | -0.42 to 0.26 |  | -0.017 | 31 | 0.93 | -0.36 to 0.33 |  |
| **Arginine** |  | 0.075 | 38 | 0.65 | -0.24 to 0.38 |  | 0.043 | 38 | 0.79 | -0.27 to 0.35 |  |
| **γ-Aminobutyric acid** |  | 0.29 | 14 | 0.27 | -0.24 to 0.69 |  | -0.061 | 14 | 0.82 | -0.54 to 0.45 |  |
| Partial correlation test was performed controlling for age and sex as covariates in depressed MDD (*N*=42). | | | | | | | | | | | |
| Significant *P*-values are shown in bold type, and underlined if they remain significant after Bonferroni correction (i.e., *P* <0.00067). | | | | | | | | | | | |
| Abbreviations: CSF, cerebrospinal fluid; dMDD, depressed (non-remitted) patients with major depressive disorder; BZD, benzodiazepine derivatives; AD, antidepressants; CI, confidence interval | | | | | | | | | | | |

**Supplemental Figure 1.** ROC curves, in which CSF EA was set as a test variable, depict the sensitivity and specificity to discriminate between two diagnostic groups. **(a)** Depressed (non-remitted) MDD vs. healthy controls. AUC of the ROC curve was 0.77 (*P*=0.0000074, 95% CI: 0.67 to 0.86). **(b)** Depressed MDD vs. remitted MDD. AUC was 0.75 (*P*=0.016, 95% CI: 0.57 to 0.93). These AUC values indicate that discrimination ability is ‘fair’.

Abbreviations: ROC, receiver operating characteristics; CSF, cerebrospinal fluid; EA, ethanolamine; MDD, major depressive disorder; AUC, area under curve; CI, confidence interval

**Supplemental Figure 2.** Scatter plot showing partial correlation between CSF isoleucine levels and daily imipramine equivalent doses of antidepressants (*r*=0.52, *df*=38, *P*=0.00056, 95% CI: 0.25 to 0.72) in the patients with major depressive disorder (*N*=42) controlling for age and sex.

Abbreviations: CSF, cerebrospinal fluid; CI, confidence interval

**Supplemental Figure 3.** Validation of EA concentrations obtained by HPLC using the CE-TOF-MS method on a subset of subjects. **(a)** Scatter plot of CSF EA levels determined by HPLC and by CE-TOF-MS (*r*=0.89, *df*=50, *P* <5×10^-18^, 95% CI: 0.81 to 0.93) with a regression line obtained using partial correlation analysis, and controlling for age and sex. **(b)** Dot plot of CSF EA levels measured by CE-TOF-MS comparing MDD with HC (*F*=8.62, *df*=1, *P*=0.0052, 95% CI: 0.000084 to 0.00045) using analysis of covariance, with age and sex as covariates.

Abbreviations: CSF, cerebrospinal fluid; EA, ethanolamine; CE-TOF-MS, capillary electrophoresis time-of-flight mass spectrometry; HPLC, high-performance liquid chromatography; HC, healthy controls; MDD, depressed (non-remitted) patients with major depressive disorder; CI confidence interval

**Supplemental Figure 4.** Signaling pathways of NAEs. FAAH mediates both pathways for synthesizing and degrading NAEs, and NAAA mainly contributes to PEA rather than other NAEs.

Abbreviations: PEA, palmitoylethanolamide; OEA, oleoylethanolamide

**References**

1. Ishidate K, Nakazawa Y. Regulation of Phosphatidylcholine (Phosphatidylethanolamine) Biosynthesis in Animal Cells. *Protein, Nucleic Acid and Enzyme* **35**, 929-940 (1990). Japanese.

2. Sun YX*, et al.* Biosynthesis of anandamide and N-palmitoylethanolamine by sequential actions of phospholipase A2 and lysophospholipase D. *Biochem J* **380**, 749-756 (2004).

3. Tsuboi K, Takezaki N, Ueda N. The N-acylethanolamine-hydrolyzing acid amidase (NAAA). *Chem Biodivers* **4**, 1914-1925 (2007).

4. Vandevoorde S, Lambert DM. The multiple pathways of endocannabinoid metabolism: a zoom out. *Chem Biodivers* **4**, 1858-1881 (2007).

5. Liu J*, et al.* Multiple pathways involved in the biosynthesis of anandamide. *Neuropharmacology* **54**, 1-7 (2008).

6. Ueda N, Tsuboi K, Uyama T. Metabolism of endocannabinoids and related N-acylethanolamines: canonical and alternative pathways. *FEBS J* **280**, 1874-1894 (2013).
